# Rubbing Powders: Direct Spectroscopic Observation of Triboinduced Oxygen Radical Formation in MgO Nanocube Ensembles

**DOI:** 10.1021/acs.jpcc.1c05898

**Published:** 2021-09-29

**Authors:** Thomas Schwab, Daniel Thomele, Korbinian Aicher, John W. C. Dunlop, Keith McKenna, Oliver Diwald

**Affiliations:** † Department of Chemistry and Physics of Materials, Paris-Lodron University Salzburg, Jakob-Haringer-Straße 2a, A-5020 Salzburg, Austria; ‡ Department of Physics, University of York, Heslington, YO10 5DD York, U.K.

## Abstract

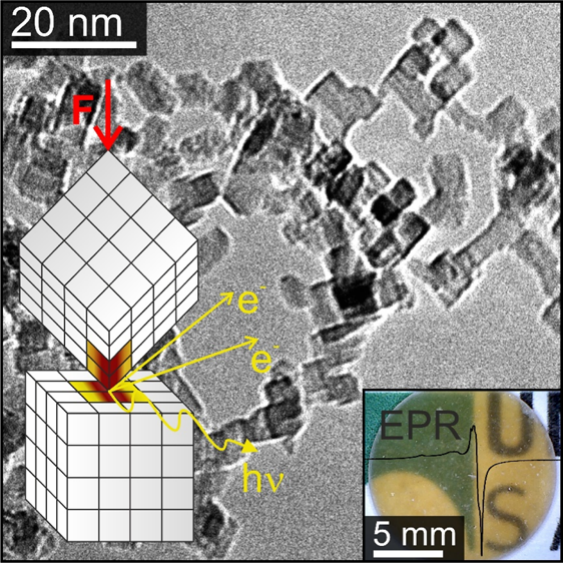

Powder compaction-induced surface chemistry in metal oxide nanocrystal ensembles is important for very diverse fields such as triboelectrics, tribocatalysts, surface abrasion, and cold sintering of ceramics. Using a range of spectroscopic techniques, we show that MgO nanocube powder compaction with uniaxial pressures that can be achieved by gentle manual rubbing or pressing (*p* ≥ 5 MPa) excites energetic electron–hole pairs and generates oxygen radicals at interfacial defect structures. While the identification of paramagnetic O^–^ radicals and their adsorption complexes with O_2_ point to the emergence of hole centers, triboemitted electrons become scavenged by molecular oxygen to convert into adsorbed superoxide anions O_2_^–^ as measured by electron paramagnetic resonance (EPR). By means of complementary UV-photoexcitation experiments, we found that photon energies in the range between 3 and 6 eV produce essentially the same EPR spectroscopic fingerprints and optical absorption features. To provide insights into this effect, we performed density functional theory calculations to explore the energetics of charge separation involving the ionization of low-coordinated anions and surface-adsorbed O_2_^–^ radicals at points of contact. For all selected configurations, charge transfer is not spontaneous but requires an additional driving force. We propose that a plausible mechanism for oxygen radical formation is the generation of significant surface potential differences at points of contact under loading as a result of the highly inhomogeneous elastic deformations coupled with the flexoelectric effect.

## Introduction

Friction between metal oxide interfaces can have important implications for surface chemistry and occurs naturally in the course of handling and compaction of nanoparticle powders. While there are a large number of triboinduced phenomena reported for metal oxides, such as tribocharging, triboelectricity, or triboluminescence,^1−6^ there exists almost no mechanistic and chemical understanding of these processes at the atomistic scale.^1^ A major challenge in characterizing contact phenomena in powders is the difficulty in precisely determining the contact area between the particles.^7^ Moreover, a variety of processes can occur between particles in contact and under powder compaction. These range from elastic and plastic deformation^8,9^ to shearing and fracture of surface elements^10,11^ such as edges and corners to coordinatively generate unsaturated surface ions of high chemical reactivity.^12−14^ Our mechanistic understanding of mechanically initiated chemical surface processes at the atomistic scale is scarce. Hence, a rational connection between triboinduced chemical activation steps including their energetics and the application of processing parameters, which induce the triboelectric phenomena, is very much needed.

Due to their simple structure and grain morphology, cubic metal oxide particles such as MgO, CaO, FeO, or NiO are extremely well-suited model systems to study tribo- and compaction-induced chemical reactions inside powders. MgO particles produced using gas-phase synthesis techniques such as chemical vapor synthesis (CVS) or flame spray pyrolysis (FSP) yield narrow particle size distributions below 10 nm in combination with a characteristic cubic particle morphology that is determined by the thermodynamically most stable (100) surfaces. The uniformity in size and shape have enabled diverse nanoparticle processes to be rationalized, including oriented attachment,^15,16^ adsorption and surface chemistry of complex organic molecules such as porphyrins,^17,18^ and the photoexcitation of specific surface structures such as nanocube corners and edges with UV light of energies *h*ν > 4 eV in great detail. Theoretically predicted energies of optical absorptions due to corners, edges, and (100) faces^19,20^ were found to be in excellent agreement with the experimental values and have even enabled us to isolate spectral contributions from buried interfaces.^21,22^ Moreover, photoexcitation studies revealed key insights into the energetics of charge separation and subsequent electron and hole trapping at surface defects.^23−25^ Structural deformation of MgO under loading is also relatively well-understood. For example, compression-induced plasticity in bulk MgO materials is related to the nucleation of dislocations, their movement, and intersections.^8,9^ Enhanced deformation rates and dislocation densities in compressed bulk MgO samples seem to also promote ion vacancy formation^26^ and triboemission of charge carriers.^27^ Meanwhile, progress in experimental techniques has allowed for addressing plastic deformation and dislocation behavior of MgO at the nanoscale.^8^ Despite these advances, important qualitative and quantitative information about the chemical species that form during these early tribochemical activation steps and the energetics of their formation is lacking.

Here, in this joint experimental and theoretical study, we find that MgO nanocube powder compaction in the dark at external pressures as low as 5 MPa (corresponding to gentle pressing or rubbing by hand) excites electron–hole pairs. Following charge separation, surface radicals are produced that are accessible to independent spectroscopic techniques such as electron paramagnetic resonance (EPR) and UV–Vis diffuse reflectance spectroscopy. We show that the resulting charge-separated configurations are equivalent to those that can be generated by UV excitation with 3–6 eV photons. Therefore, gentle rubbing of a powder is sufficient to surprisingly access energetically excited states and produce reactive surface species. These phenomena could find applications in the polishing and chemical treatment of surfaces as well as novel tribovoltaic and tribocatalytic^6^ materials for the harvesting of low-level mechanical energy. There is a so far unexplored opportunity in the field of intergranular surface chemistry, where we anticipate that our findings would be of interest for new approaches in chemical and cold sintering as well as for the design of novel composites that can be polymerized at room temperature to achieve homogeneously distributed nanoparticles.

This study is structured as follows: in the first part, we describe the experimental findings (structure characterization and spectroscopy) we obtained in the course of MgO nanocube powder compaction-induced charge separation. We compare the outcome of this process in the dark with paramagnetic states that originate from UV- excitation to conclude the nature of local defects and the energies required to form them. Then, in the second part, we analyzed different local particle–particle configurations and present density functional theory (DFT) results related to optimized structures of ionized donor and negatively charged acceptor species. Finally, we discuss potential processes of energy dissipation in the regimes of elastic and plastic deformation to explain the compaction-induced charge separation effects observed.

## Materials and Methods

### CVS and Powder Annealing Protocol

MgO nanocrystals obtained from CVS correspond to the controlled combustion of Mg metal vapor in the presence of oxygen under reduced pressure. Further information is provided in refs (21), (22), and (28). The employed reactor system consists of two concentrically arranged quartz glass tubes mounted inside a cylindrical tube furnace. The inner tube hosts ceramic crucibles filled with Mg metal grains (99.98% trace metal basis, Alfa Aesar). Heating up to 913 K leads to sublimation and guarantees a sufficiently high metal vapor pressure. An inert argon flow [Ar 5.0, *Q*(Ar) = 1250 sccm] guides the Mg vapor to the end of the inner glass tube, where the argon/metal vapor mixture gets into contact with molecular oxygen [O_2_ 5.0, *Q*(O_2_) = 900 sccm] streaming through the outer tube. At this position, a highly exothermic reaction in the presence of oxygen is initiated, which leads to the formation of MgO nanoparticles from homogeneous nucleation in the gas phase. Short residence times of resulting nuclei in the hot reaction zone (<2 ms),^29^ guaranteed by the Ar flow and continuous pumping down to *p* = 50 ± 2 mbar, prevent undesired particle coarsening and coalescence. The nanoparticle agglomerates are collected in a stainless-steel net at room temperature downstream the reactor. Total pressure, gas flows, and furnace temperature are kept constant during the entire period of particle collection.

Powder annealing was performed in dedicated fused silica cells attached to a high-vacuum rack, which allows for pressures as low as *p*(O_2_) < 10^–5^ mbar and defined gas atmospheres. Sample heating up to 1123 K is described by a stepwise temperature increase of 100 K with heating rates of 5 K/min (room temperature up to 373 K) and 10 K/min (rest of the protocol), respectively. The next heating step is initiated as the pressure falls below *p*(O_2_) < 9 · 10^–6^ mbar. Admission of pure oxygen [*p*(O_2_) = 10 mbar] was performed at 1123 K and dwelled for 10 min. After subsequent evacuation to *p*(O_2_) < 9 . 10^–6^ mbar, the sample was heated to the final temperature (1173 K, *r* = 10 K/min) and dwelled for 60 min prior to cooling down to room temperature.^17,30^

### Powder Compaction

Powder compaction was performed via cold uniaxial pressing resulting in a regular disk-shaped specimen. A defined mass of powder (*m* = 150 ± 10 mg) was transferred into the cavity (*d* = 13 mm) of a compaction tool (FTIR Pellet Dies, Specac) and uniaxially compressed with a hydraulic press (Atlas manual hydraulic press 15T, Specac) under an applied pressure between p = 1 MPa and 74 MPa that was dwelled for 1 min to obtain green compacts in a controlled and reproducible way. To minimize the amount of water adsorption, powder transfer and compaction was performed inside glovebags filled with Ar at room temperature.

### X-ray Diffraction

Continuous scan powder X-ray diffraction (XRD) data were collected at room temperature in the coupled Θ–Θ mode on a Bruker D8 Advance with a DaVinci design diffractometer, having a goniometer radius of 280 mm and equipped with a fast-solid-state Lynxeye detector and an automatic sample changer. Powder samples were prepared on a single-crystal silicon zero-background sample holder. Data acquisition was performed using Cu Kα_1,2_ radiation (λ = 154 pm) between 5 and 80.5° 2Θ with a step size of 0.02° and opened divergence and antiscatter slits at 0.3 and 4°, respectively. A primary and secondary side 2.5° Soller slit was used to minimize axial divergence and a detector window opening angle of 2.93° was chosen. Data handling and qualitative phase analysis was performed with the Bruker software DIFFRAC.EVA V2.1. Crystallite domain sizes were derived from the integral reflection width by applying the Scherrer equation to the (200) main diffraction feature.

### Transmission Electron Microscopy

Transmission electron microscopy (TEM) data were acquired using a JEOL JEM-F200 transmission electron microscope operating at 200 kV equipped with a cold field emission electron source. TEM images were recorded using a TVIPS F216 2k by 2k CMOS camera to access morphological and structural information. Evaluation of images acquired during TEM analysis was performed with either ImageJ (V1.52a) or the EM Measure software from TVIPS.

### Nitrogen Sorption

N_2_-sorption analysis was performed at 77 K and the specific surface area was calculated by applying the model of Brunauer–Emmett–Teller (BET). Prior to sorption measurements with an ASAP 2020 adsorption porosimeter from Micromeritics GmbH, each sample was degassed under vacuum at 573 K for 3 h. The BET surface area (*S*_BET_) was evaluated using adsorption data in a relative pressure range *p*/*p*_0_ of 0.06–0.21. Under the assumption of equally sized and nonporous cubic particles, eq 1 provides an average value for the particle size (*d*_BET_), derived from the integral sample volume, where ρ depicts the theoretical density of MgO.^31^ Additionally, pore size analysis was performed by applying the BJH model to the recorded sorption isotherms.
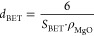
1

### EPR Spectroscopy

X-band EPR measurements were performed on a Bruker EMXplus-10/12/P/L spectrometer equipped with an EMX^Plus^ standard cavity and using an NMR teslameter that allows for accurate determination of resonant field values. MgO green compact fragments were placed inside a Suprasil quartz glass tube (*d* = 5 mm) and connected to the EPR high-vacuum line with base pressures as low as *p*(O_2_) < 10^–5^ mbar, which also allows for in situ thermal treatment up to 1173 K as well as addition of pure gas atmospheres [*p*(O_2_)] combined with UV-excitation. Polychromatic illumination of reannealed powder compacts [*p*(O_2_) < 10^–5^ mbar and *T* = 1173 K] at the spectrum acquisition temperature was carried out using a 300 W Xe-arc lamp equipped with a water filter to exclude IR contributions from the excitation spectrum. EPR spectra were recorded at 10 K using a waveguide cryogen-free system (Oxford Instruments). For spectrum acquisition, a microwave power of 2 mW, at a field modulation frequency of 100 kHz with an amplitude of 1 G, was chosen.

The experimental spectra were simulated using the Matlab-based computational package EasySpin,^32^ which is freely available. Spin systems of axial and rhombic *g*-tensor symmetries were modeled using Gaussian and/or Lorentzian line shapes. The best fit was obtained after performing a least-squares fitting routine of multiple component EPR spectra. The aim of this approach was to perform a qualitative assignment of species, based on a profound knowledge about oxygen-related paramagnetic species that are well-known to form on the surface of MgO nanoparticles.^33−35^ Further details of the simulation are provided in Supporting Information.

### Theoretical Calculations

To calculate the total energies of the various donor and acceptor configurations, we employ a quantum mechanical embedded cluster approach, which combines quantum mechanical and pair-potential levels of theory to describe large aperiodic systems such as MgO nanocrystals.^22,36,37^ A cluster of atoms centered near a region of interest (e.g., the large red and green spheres in Figure 5b for the oxygen-terminated corner-terrace feature) are treated quantum mechanically with other atoms (small red and green sphere in Figure 5b) described using a polarizable shell model potential.^38^ Mg and O ions in the quantum region are described at the all-electron level using a Gaussian 6-311G* basis set and the B3LYP hybrid density functional^39^ (a 6-311+G** basis is used for H when modeling OH). To prevent spurious spilling of the wave function from the quantum cluster into the classically modeled regions, the first and second nearest-neighbor Mg are modeled using a semilocal effective core pseudopotential possessing no associated basis functions (gray spheres in Figure 5b). The total energy of the quantum cluster, in the presence of the electrostatic potential produced by all surrounding classical ions, is obtained by solving the Kohn–Sham equations. The geometry of the entire system is optimized self-consistently using the BFGS algorithm until forces on atoms are less than 0.01 eV Å^–1^. This method is implemented in the GUESS code interfaced with the Gaussian 03 code for the quantum mechanical part of the calculation.^36^ The accuracy of this approach for low-coordinated features in MgO nanopowders has been verified in comparison with experimental results in previous studies.^21,40^ The approach also gives a good description of the molecular species studied here. In the gas phase, for O_2_, *d*_O–O_ = 1.21 Å (exp: 1.21 Å) and an electron affinity (EA) of 0.29 eV (exp: 0.44 eV), for O_2_^–^, *d*_O–O_ = 1.35 Å (exp: 1.34 Å), and for OH^–^, *d*_O–H_ = 0.98 Å (exp: 0.97 Å).

## Results and Discussion

### Structure and Microstructure of MgO Nanocrystal Compacts

**Figure 1:**
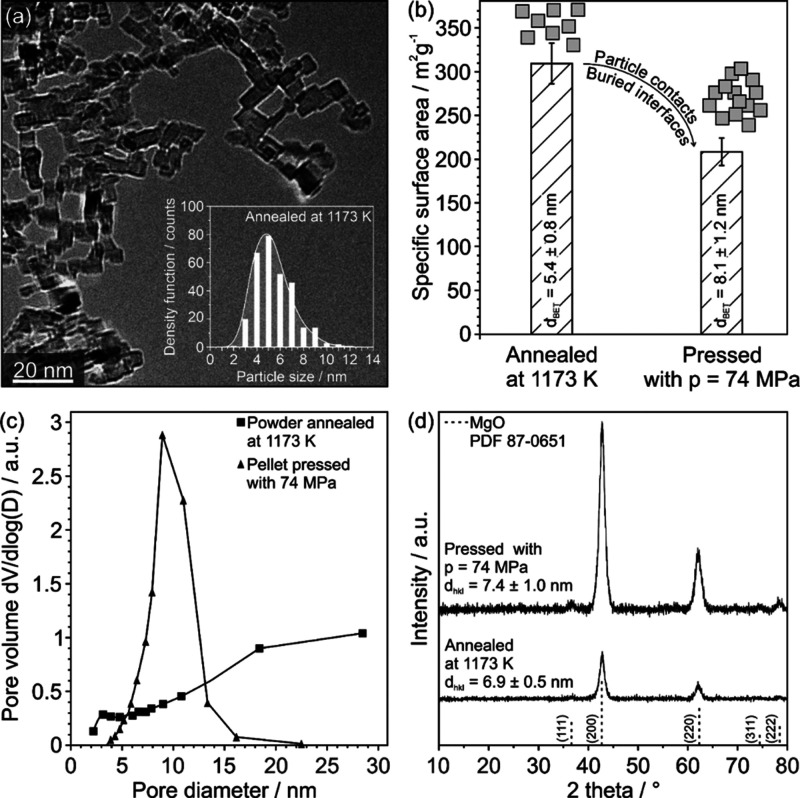
Transmission electron micrograph (a) of MgO nanocubes after annealing at 1173 K and prior to compaction, with a particle size distribution plot as the inset. Specific surface areas (b) and pore size distribution functions (c) were accessed with nitrogen sorption analysis before and after uniaxial compaction with 74 MPa. XRD patterns of 1173 K annealed powder samples prior to and after compaction are provided in (d).

Compaction-induced changes in the structural properties of annealed MgO nanocube powders were characterized using TEM, XRD, and N_2_-sorption analysis (Figure 1). TEM (Figure 1a) shows highly dispersed powders of MgO nanocubes exhibiting sharp edges and corners. The powder agglomerates are composed of individual single-crystalline MgO nanocubes that are loosely attached to each other due to electrostatic interactions. Powder compaction (*p* = 74 MPa) reduces the surface area of the nanocube powder by roughly 30%, from 300 to 200 m^2^/g (Figure 1b). The change in the surface area is associated with the formation of a mesoporous nanoparticle network showing a pore size maximum at approximately 10 nm (Figure 1c). On the basis of powder XRD data (Figure 1d), we can rule out that MgO nanocube powder compaction induces changes in the crystal phase and crystallite size. Moreover, there are no strain effects in the XRD pattern, which would give rise to the shift of MgO-specific diffraction features, observable. Broad reflection features at 2Θ positions that are characteristic for the cubic rock-salt structure of MgO nanocrystals are observed before and after powder compaction. The integral reflection widths remain unaltered and consistent with an average crystallite domain size of *d*_XRD_ = 7 nm.

### Optical Property Changes and Charge Separation

**Figure 2:**
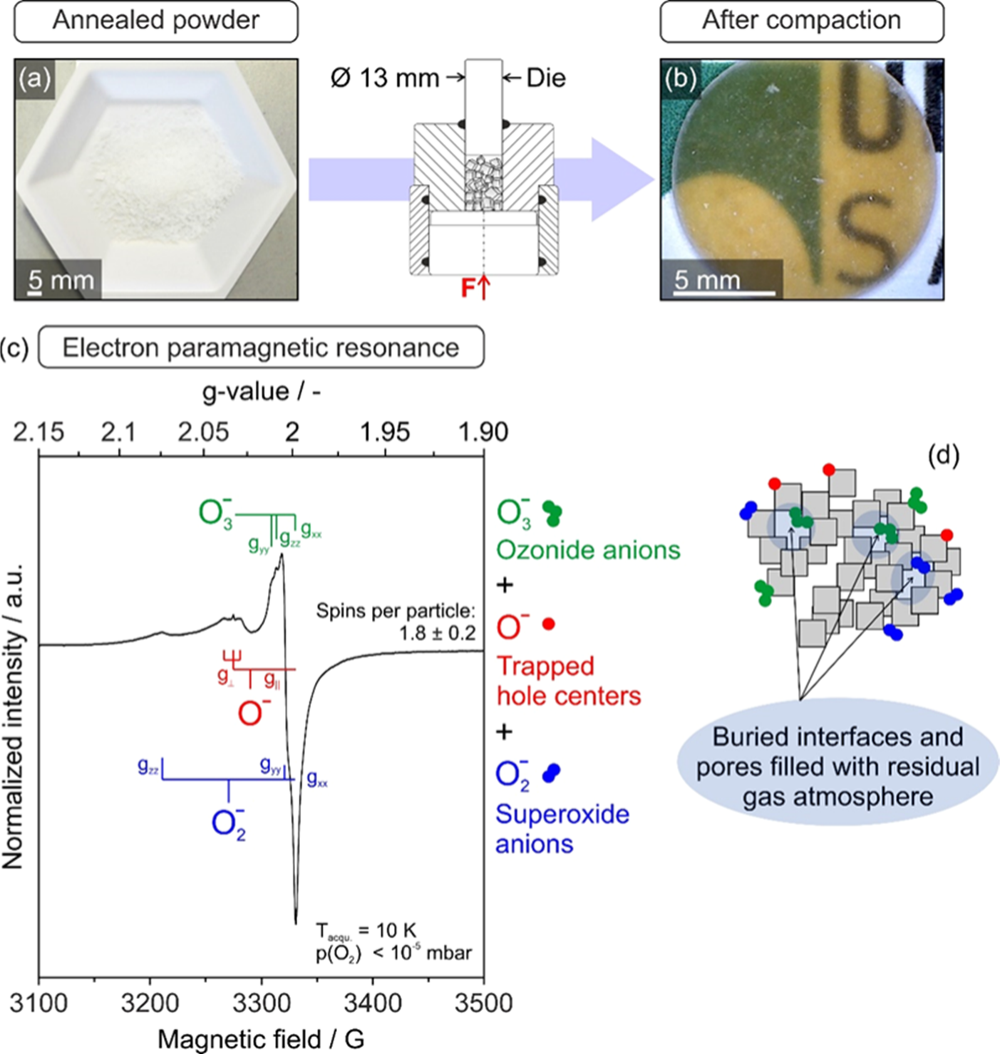
Digital photographs of MgO nanocube powder (a) prior to and (b) after uniaxial compaction (*p* = 74 MPa) revealing a pressure-induced color change from white to brownish. EPR spectrum of fractured compacts acquired under dynamic high-vacuum conditions [*p*(O_2_) < 10^–5^ mbar] is plotted in (c). Schematic drawing in (d) illustrates different types of adsorbed paramagnetic oxygen species, which emerge upon compaction and remain partly encapsulated inside the closed pores.

MgO nanocrystal powder compaction alters the optical powder properties (Figure 2a→b) in a similar way as it has previously been observed in the course of UV-excitation experiments (*h*ν = 4.6 eV and λ = 270 nm) of MgO nanocube powders in an oxygen atmosphere.^35^ Transformation of the optical absorption properties of the wide band gap material (*E*_g_ = 7.8 eV)^41^ from a white scattering powder (Figure 2a) to a brown/yellow translucent MgO nanoparticle compact (Figure 2b) is attributed to the emergence of paramagnetic oxygen radicals. Figure 2c shows a typical EPR spectrum that was acquired on brownish MgO compact fragments.

The complex envelope of the EPR signal (Figure 2c) covers the magnetic field range where oxygen radicals typically resonate.^14,34,42−44^ The powder spectrum simulation reveals the presence of three contributing species with *g*-parameters that are consistent with oxygen radical sites (O^–^, O_2_^–^, and O_3_^–^) (Table 1). A detailed temperature and microwave power dependence of the EPR signal shape (not shown) was included for this analysis. A rough quantification revealed about 2 spins per particle on average.

**Table 1:** *g*-Parameter Values of Oxygen Radicals Detected on Annealed MgO Nanopowders after UV Excitation,^33−35^ in Comparison with Those Isolated after Compaction of Annealed MgO Nanocube Powders within This Study (Figure 2)

paramagnetic species	mean *g*-value			references
trapped hole centers O^–^	*g*_⊥_	*g*_∥_		
	2.0359 ± 0.0001	2.0021 ± 0.0002		literature
	2.0337 ± 0.0015	1.9995 ± 0.0016		this study
superoxide anions O_2_^–^	*g*_*zz*_	*g*_*yy*_	*g*_*xx*_	
	2.0840 ± 0.0028	2.0075 ± 0.0007	2.0023 ± 0.0000	literature
	2.0744 ± 0.0021	2.0053 ± 0.0015	1.9995 ± 0.0016	this study
ozonide anions O_3_^–^	*g*_*yy*_	*g*_*zz*_	*g*_*xx*_	
	2.0163 ± 0.0015	2.0116 ± 0.0011	2.0020 ± 0.0005	literature
	2.0126 ± 0.0016	2.0100 ± 0.0013	1.9995 ± 0.0016	this study

The oxygen radical species that form upon compaction (Figure 2) have also been produced by UV-excitation of nonconsolidated MgO nanoparticle powders and were interpreted in terms of photoinduced charge separation.^24,33−35^ Although the individual EPR-signal components are broadened in comparison with the UV-excitation results,^33−35^ the *g*-parameters are in very good agreement with the earlier reported EPR signatures of trapped hole centers (O^–^-signal with axial symmetry) and superoxide and ozonide anions (O_2_^–^ and O_3_^–^ -both three-signal components) (Tables 1 and S1 of Supporting Information). The signal broadening observed is attributed to two major effects:

(i) powder compaction leads to higher concentrations of paramagnetic defects which can emerge in close vicinity to each other and undergo spin-exchange interactions;
(ii) a fraction of paramagnetic oxygen molecules remains trapped inside the internal voids and closed pores of the compacts (Figure 2d). At cryogenic temperatures, they condense at the pore walls and additionally contribute to spin-exchange-induced signal broadening.

Previous studies on nonconsolidated loose nanoparticle powders have shown that pumping to pressures as low as *p*(O_2_) < 10^–5^ mbar at room temperature leads to the decomposition of the paramagnetic O_3_^–^ adduct into its components O^–^ and O_2_. In the present study, however, we find that the ozonide ions remain after pumping at room temperature, which serves as clear evidence for their encapsulation in the internal pores (in fact, further experiments supporting this hypothesis are provided by Figures S1 and S2 in Supporting Information).

Deviations in the EPR signatures of oxygen radicals obtained by compaction versus UV-excitation of uncompacted powders are in the range Δ*g* = ±10^–3^ or less. This can be attributed to the altered microstructure of the MgO nanocube ensembles after powder compaction. New particle contacts and interface configurations emerge and give rise to qualitatively new adsorption sites for O_2_^–^ and O_3_^–^ complexation that can participate in the pressure-induced charge separation process. Moreover, at the EPR acquisition temperature of *T*_acqu._ = 10 K, residual oxygen and traces of water, which unavoidably remain trapped as a result of the compaction step in the nitrogen-filled glovebag, also produce slightly altered local electronic environments around the paramagnetic adsorbates (O^–^, O_2_^–^, and O_3_^–^).

**Figure 3:**
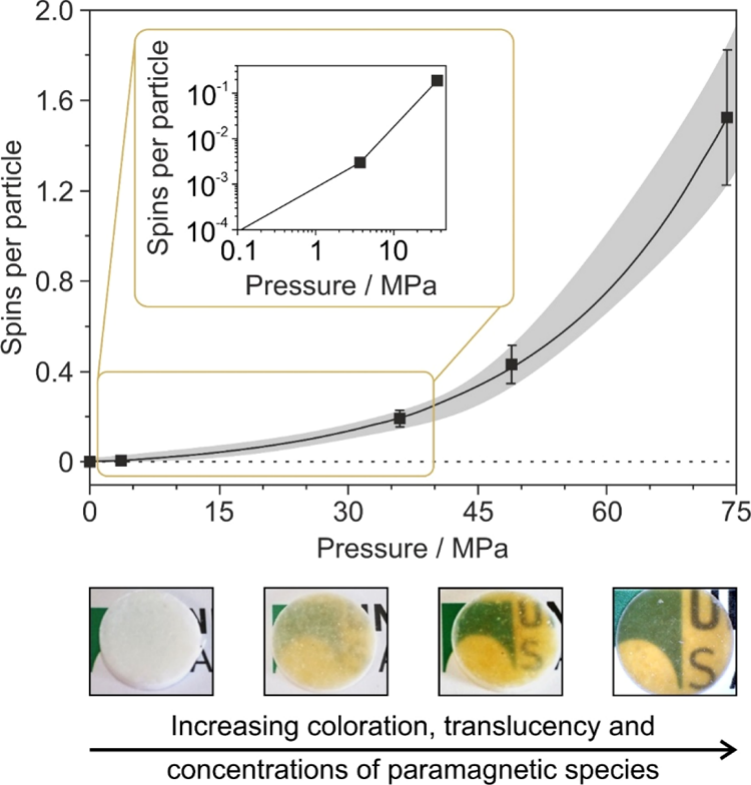
Charge separation yield determined by EPR on powder compacts as a function of the uniaxial pressure applied. The digital photographs annotated to the different data points clearly reveal the optical property changes the compacts undergo with increasing pressure. Typical quantities of MgO nanocube powders are 75 mg, corresponding to roughly 10^17^ nanocubes inside the volume assessed by EPR spectroscopy.

There are essentially three effects that can be observed on the MgO nanocube powder compacts after application of uniaxial pressure in the range between 5 MPa (pressure generated by hand) and 75 MPa: charge separation (Figure 2c), color change, and an increase in translucency of the compact (Figure 2b). Note that pressures, which can be already generated by hand (approximately 2–5 MPa), are effective to produce charge separation and, thus, paramagnetic surface radicals at a number of the order of 10^14^ spins (Figure 3). Clearly above the threshold of detectable number of spins inside the continuous wave X-band EPR cavity used (with *N* > 10^11^), this concentration corresponds roughly to 10^–3^ spins per particle. The application of higher pressures and, concomitantly, higher values for the compressive engineering stress (σ ≥ 37 MPa) by a hydraulic press increases the compacts’ translucency and -at the same time -the intensity of its yellow/brown color (Figure 3). Throughout the entire range of external pressures investigated, the shape of the EPR signals measured remained constant, indicating a constant relative abundance of the paramagnetic species involved and thus no significant changes in the underlying mechanism of charge separation.

### Compaction Versus UV Excitation-Induced Charge Separation

**Figure 4:**
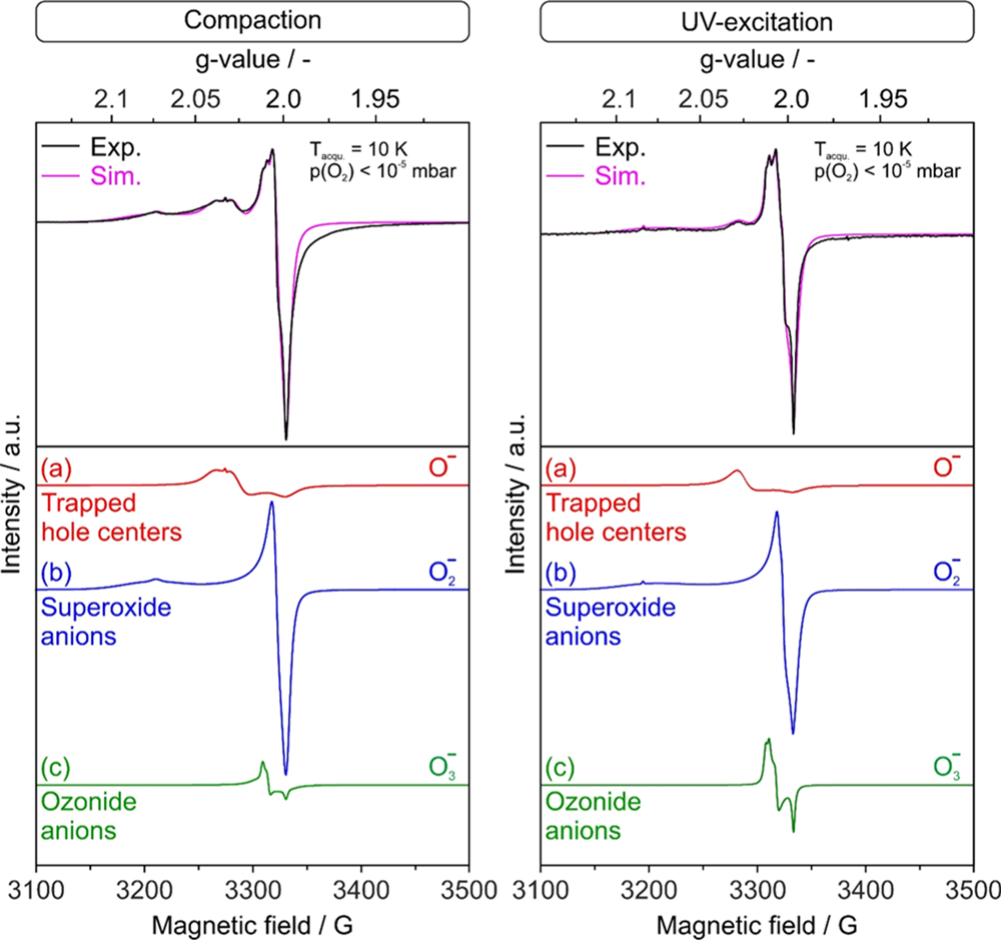
EPR powder spectrum analysis related to oxygen radicals detected on MgO samples directly after compaction (left panel) and after reannealing of the fractured MgO powder compacts, followed by subsequent polychromatic excitation in an O_2_ atmosphere (right panel). The *g*-parameters of the best-fit results together with further simulation details are listed in Table S1 of the Supporting Information section.

For validation of the hypothesis that the altered microstructure of the MgO nanocube ensemble gives rise to the small variations in the *g*-parameter values, we also included UV-excitation experiments in this study (Figure 4).^25^ For this purpose, a powder compact, such as the one characterized along Figures 2 and 4 (left panel), was reannealed in vacuum and up to *T* = 1173 K. All paramagnetic species are annihilated and the corresponding EPR spectrum was found to be exempt from any type of resonance signal (flat line, not shown). Subsequent UV excitation in an O_2_ atmosphere generates oxygen radicals with an EPR powder spectrum (Figure 4, left), which is essentially identical to that acquired after powder compaction in the dark (Figure 4, right).

A systematic investigation of UV-excitation effects on MgO was performed in earlier work^24^ and revealed that a minimum photon energy of 4.6 eV is required to generate and dissociate surface excitons at MgO nanocube edges and corners.
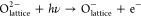
2

While the hole component remains trapped at lattice oxygen anions from the MgO nanocube surfaces and interfaces, the photogenerated electron becomes scavenged by molecular oxygen.
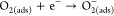
3

As an additional process, photoexcitation with a threshold energy of 4.0 eV was found to initiate an oxygen adsorption-assisted process of ozonide (O_3_^–^) and superoxide (O_2_^–^) anion formation.
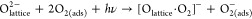
4

The photoexcitation at 4.0 eV is directly linked to a previously reported photoluminescence emission process at 2.5 eV that was observed for particle systems with an appreciable concentration of solid–solid interfaces.^21^ Molecular oxygen quenches the emission process completely. Consequently, the surface and interface sites involved are accessible to gas-phase species at the outer surfaces of the porous particle compact.

### Particle–Particle Configurations and the Feasibility of Charge Separation

As described above, there is clear experimental evidence that compression induces electron transfer between donor and acceptor features in the nanopowder, resulting in a final charge-separated configuration very similar to that obtained by UV-excitation. The surface-adsorbed O_2_ molecule is clearly identified through its EPR spectra (i.e., an O_2_^–^ species) as the electron-trapping species (acceptor). The hole-trapping species (donor) is thought to be a low-coordinated oxygen ion (either at a free corner or a point of contact such as a corner-terrace feature). One question that emerges is whether the formation of new structural features at particle–particle points of contact by compression could be sufficient to explain the observed charge transfer by significantly reducing the energy cost for electron–hole separation. Here, we attempt to answer that question by calculating total (ground state) energy differences between candidate donor and acceptor features in the nanopowder using DFT and the embedded cluster method (see the Materials and Methods). Energy differences calculated in this way correspond to a quasi-static equilibrium and as such do not account for nonequilibrium processes that may occur during compression that may directly lead to excitation of phonons or electrons.

**Figure 5:**
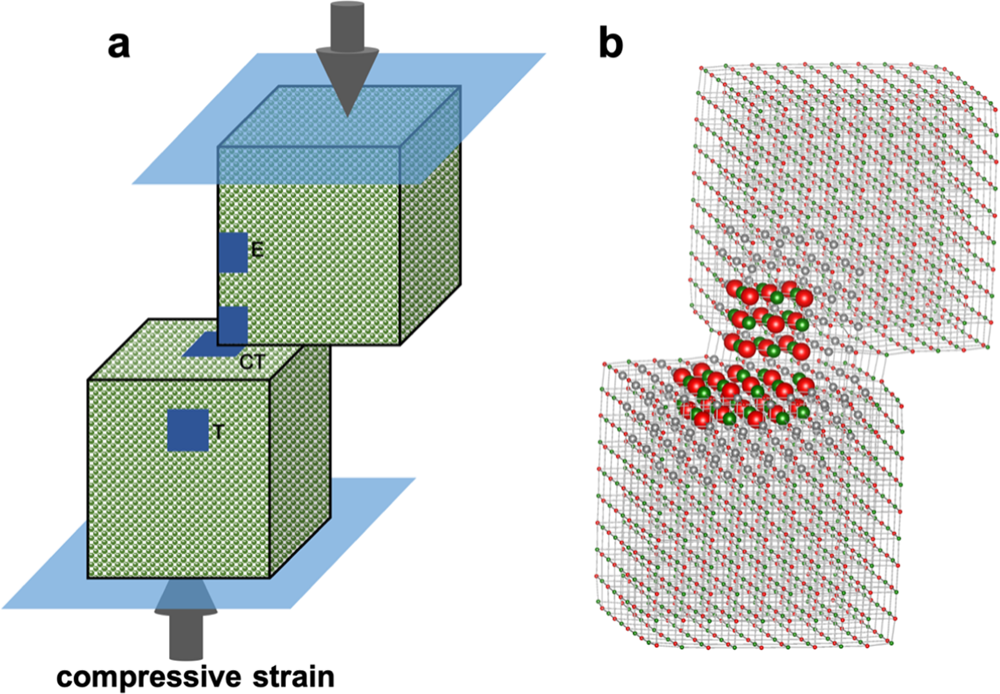
(a) Schematic showing the model we employ to model interfaces in MgO nanopowders with several local features highlighted: oxygen-terminated corner-terrace (CT), edge (E), and terrace (T). (b) Embedded cluster method setup for the oxygen-terminated CT feature. Large red and green spheres represent atoms treated at the quantum mechanical level, small red and green spheres represent atoms treated using the polarizable shell model, and large gray spheres represent the Mg atoms modeled using a semilocal effective core pseudopotential to prevent spurious spilling of the wave function from the quantum cluster into the classically modeled regions. The embedded cluster method setups for the other features of interest are similar but with the quantum region centered on the relevant feature.

Figure 5 shows the model we employ consisting of two MgO nanocubes in contact and several local features at or near a point of nanocrystal contact are highlighted: oxygen-terminated corner-terrace (CT), terrace (T), and edge (E). This model is based on earlier electron microscopy studies of typical features seen in MgO nanopowders and our previous theoretical work on their corresponding optical properties.^21,40,45^ For the donor features, we consider two possibilities for which the ionization energy (IE) should be relatively low including the oxygen-terminated corner-terrace and a surface-adsorbed hydroxyl group (OH^–^). For the former, we consider the interface with and without compressive strain (we apply 4% compressive strain as indicated in Figure 5a to investigate the effect this may have). For the acceptor features, we consider O_2_^–^ adsorbed at the surface of MgO nanocrystals (T or E) or near a point of contact between nanocrystals (CT).

**Figure 6:**
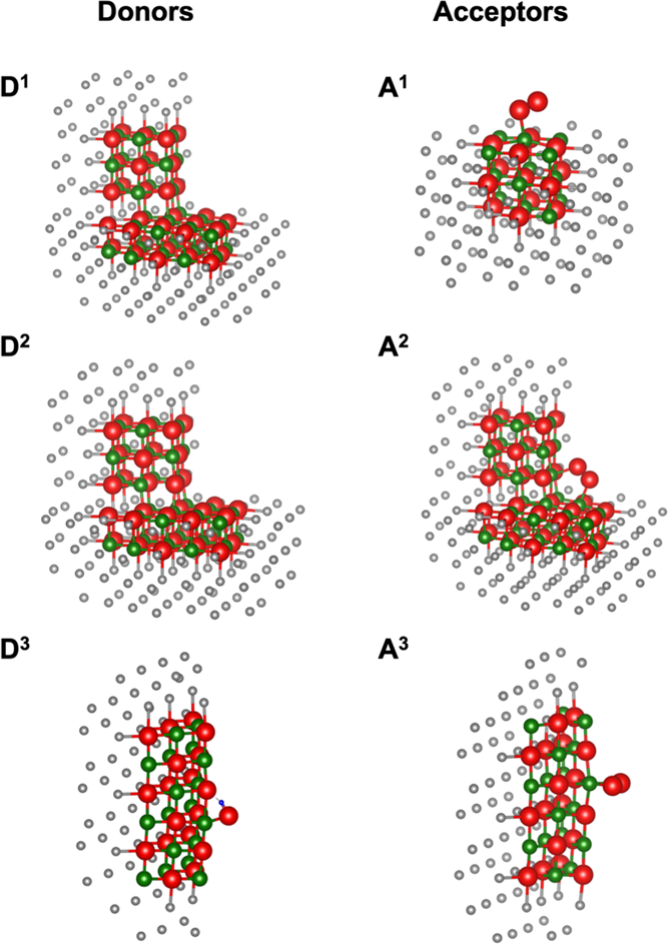
Optimized structures of ionized donor and negatively charged acceptor features in the MgO nanopowder: oxygen-terminated corner-terrace (D^1^), oxygen-terminated corner-terrace under 4% compressive strain (D^2^), hydroxyl adsorbed on an edge (D^3^), oxygen molecule adsorbed on a terrace (A^1^), oxygen molecule adsorbed near the oxygen-terminated corner-terrace (A^2^), and oxygen molecule adsorbed on an edge (A^3^).

Figure 6 summarizes the optimized structure of the ionized donor and negatively charged acceptor features we have considered. The calculated IE for the oxygen-terminated corner-terrace (D^1^) is 4.9 eV. Application of a 4% compressive strain, followed by full geometry optimization is found to reduce the length of the O corner–Mg terrace bond by about 3% (D^2^). However, this leads to only a 0.1 eV reduction in the IE suggesting by itself that local strain of bonds due to compression is not likely responsible for inducing charge transfer. The calculated IE for the hydroxyl adsorbed on an edge is 3.2 eV. Turning now to the acceptor features, we find the EA of the oxygen molecule adsorbed at the terrace is 2.6 eV (A^1^), at the oxygen-terminated corner-terrace is 1.5 eV (A^2^), and at the edge is 2.9 eV (A^3^).

To compare the feasibility of the various possible charge-transfer processes, we summarize the energy difference Δ = IP – EA for the donor–acceptor pairs in Table 2. One would expect that charge transfer would be favored for donor–acceptor pairs with small or negative energy differences Δ. However, the majority of the prospective processes we have considered have relatively large Δ. The transfer of an electron from a hydroxyl adsorbed at an edge to an oxygen molecule adsorbed at an edge (D^3^–A^3^) has the lowest Δ of 0.3 eV. However, this is unlikely to explain the experimentally observed compression-induced charge separation as the hole that is left on the donor remains localized on the hydroxyl oxygen. If this were the correct charge-separated state, one would see evidence for it in the form of hyperfine splitting in the EPR spectra, but this is not seen experimentally. Of the charge-transfer processes directly involving a point of contact, the lowest energy difference is for electron transfer from a strained oxygen-terminated corner-terrace to an oxygen molecule adsorbed at an adjacent edge (D^2^-A^3^) with Δ = 1.9 eV.

**Table 2:** IE, EA, and Energy Difference Δ for Various Donor–Acceptor Pairs (See Figure 6 for the Definition of Feature Labels)^a^

donor–acceptor	IE (eV)	EA (eV)	Δ (eV)
D^1^–A^1^	4.9	2.6	2.3
D^1^–A^2^	4.9	1.5	3.4
D^1^–A^3^	4.9	2.9	2.0
D^2^–A^1^	4.8	2.6	2.2
D^2^–A^2^	4.8	1.5	3.3
D^2^–A^3^	4.8	2.9	1.9
D^3^–A^1^	3.2	2.6	0.6
D^3^–A^2^	3.2	1.5	1.7
D^3^–A^3^	3.2	2.9	0.3

a The calculated energy differences provide a thermodynamic measure of the possibility of charge transfer but the kinetic barriers could be higher.

The analysis performed here at the quantum level points to a number of local contact configurations that would facilitate the separation and consecutive charge transfer. However, while all the energy costs computed are below 4.6 eV (the energy threshold seen in the UV-excitation experiments, which serves here as a reference to powder compaction-induced charge separation), they still will require energies ≥ 1.9 eV. Thus, in the absence of additional driving forces, the local defect geometries alone cannot explain the spontaneous occurrence of charge separation.

One example of a plausible additional driving force is the flexoelectric effect. The flexoelectric effect can occur in essentially all types of insulating materials (ceramics and polymers) and is characterized by a linear coupling between a local strain gradient and polarization. In recent work Mizzi et al.^46^ addressed the question whether flexoelectric potential differences that are induced by inhomogeneous strain at nanoscale asperities can drive tribocharge separation and transfer. Based on Hertzian and Johnson–Kendall–Roberts contact models for elastic deformation, their analysis revealed that large strain gradients can give rise to surface potential differences in the range of ±1–10 V. Such inhomogeneous strain effects with their intrinsic asymmetry can emerge on a variety of solid parts in movement, such as particles during milling and inside powders during compaction. In fact, MgO nanocubes and their aggregates exhibit a large abundance of such asperities in the form of nanocube edges, step edges, or corners. Upon powder compaction, all of these surface features get in contact with surface elements present on adjacent particles. Based on the computed energies mentioned above, the typical potential differences that can be generated by the flexoelectric effect could be sufficient to induce charge transfer under compaction.

### Powder Compaction and Plastic Strain

We now turn to consider whether plastic strain associated with the nonequilibrium powder compaction process itself can in principle provide the necessary energy to facilitate charge separation. When the MgO powder is compacted, the relative density changes from 1.3 to 25%, much lower than the theoretical maximum random packing density of 63.7%.^47^ This low density may arise from the tendency of the cubic MgO particles to stack and form elongated clusters (see TEM image Figure 1a). Assuming that the compacted powder consists of perfectly packed and aligned cubic particles, it is possible to get an order of magnitude estimate of the local stresses and therefore deformation energies in the particles during loading. Assuming that all particles are loaded identically, we can estimate the effective loaded area per particle as being
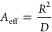
5where *R* is the particle size and *D* is the relative density. This can be then used to estimate the local normal force on each particle as a function of the macroscopic stress
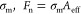
6

Perfectly aligned particles would therefore have a local stress which scales with σ_m_/*D*. For plastic compaction one expects the pressures scaling as three times the yield stress,^48^ our estimate of ∼300 MPa for the powder compressed to 74 MPa is well above 3 times the yield stress of the MgO particles at room temperature.^9^ Despite the simplifications, such a loading implies that plastic yield in the particles is indeed likely to occur in the powder compacts for the experimental loads and densities. The loading experienced by each particle will depend of course on the detailed interactions between the surrounding particles. This is determined by the degree of alignment with nonlocal processes such as buckling of larger particle clusters, as well as frictional sliding modifying the local stress states in a complex manner. It is possible that the stresses can locally be much higher than estimated. It remains now to estimate the local energies involved in plastic compaction. During the densification from 1.3 to 25% relative density, it can be expected that a large amount of macroscopic deformation arises due to particle and cluster rotation, bending, and buckling. This makes it difficult to estimate local plastic strains, a more detailed analysis would require discrete element calculations such as those done by ref (48). We can, however, calculate the required plastic strain to give the 4.6 eV required for charge separation. By estimating the local plastic work on each particle from the normal force calculated above, we calculate a minimum plastic strain of only 0.7% per particle to give enough energy per particle for charge separation (or lower if one considers donor–acceptor species associated with points of contact as discussed in the previous section). If this strain is localized inside the particle, at the corners or edges, for example, it would thus be possible for plastic strain during compaction to give rise to charge separation and the resultant color changes observed.

Recent advances in in situ TEM measurements in combination with the use of nanocubes of MgO smoke and complementary molecular dynamics (MD) simulations revealed details on elastic and plastic deformation at the level of individual particles.^8,9^ For MgO nanocubes at room temperature, Amodeo et al.^8,9^ reported an elastic behavior for strains up to 11%. Nucleation of the first dislocations was experimentally observed at true compressive stress values around 1 GPa and above. In the course of their combined experimental and theoretical study, the authors identified dislocation-related size effects. MD results indicate that in MgO nanocubes smaller than 8 nm, deformation occurs through dislocation nucleation at (100) surfaces, corners, and edges. Evidence for the interaction between dislocations was primarily obtained for larger cubes. Despite these advances, for nanoparticles present in a powder, we still are limited in the fact that the contact area between the particles, which will determine local stresses, is difficult to define and is strongly dependent on the local microstructure of the powders.

When a powder of particles is compacted under pressure, the point contacts between the particles flatten to areal contacts. In such a porous compact, any transmission of force takes place mainly through this contact region. From the present study, it is clear that the low density of the powder compact, as well as the heterogeneity in pore sizes (presence of mesopores, Figure 1c, and large macropores), and the microstructure are complex. We see stackings of cubes (Figure 1a) which could potentially lead to more complex clustering at higher length scales. Upon compression, it is likely that locally certain clusters or stacks would be placed under complex loading (bending, torsion, and local rearrangement due to fracture). Although regions of the microstructure will be loaded elastically, it is clear that local plasticity and/or fracture bond breaking must occur for compaction. The resultant behavior will be a combination of elastic and plastic deformation which as we have seen from the literature can both give rise to charge separation.^46^

Here, it is also important to note that compaction-induced charge separation was observed on nanocrystalline MgO with dehydroxylated particle surfaces and in pristine environments. The next logical step will be to explore the impact of the environmental gas atmosphere, such as the presence of water vapor on charge separation yield and to expand this investigation to other metal oxides of comparable dispersion.

As a summary of the here-discussed results, we can state that our experimental investigations demonstrate that MgO powder compaction induces the formation of oxygen radicals by charge separation essentially indistinguishable from those generated by UV-excitation of the same compacted material. The mechanism for charge separation by UV- excitation is well-understood, involving the photoexcitation of low-coordinated anion features leading to electron transfer to acceptor species such as adsorbed oxygen molecules. This process requires photon energies of around 4.6 eV or higher.^25^ This suggests that compression alone is likely to be inducing a very similar charge-transfer process; however, the mechanism is not currently known.

To provide some insights into possible mechanisms, we have performed a number of DFT calculations to assess the electronic properties of features at points of contact between nanocrystals as well as at dislocations that may be generated under loading, facilitating plastic deformation. We have also provided estimates of the energy that may be released by compression-induced plastic deformation. The DFT calculations consider the electronic properties of various features in local equilibrium to assess whether their structure alone is sufficient to induce charge transfer. While the answer is negative, we discuss how the flexoelectric effect that has recently been invoked to explain tribocharge separation and transfer at nanoscale asperities can provide an additional driving force for such charge-transfer processes. We also show that it is likely that under compression, some degree of plastic deformation may take place involving the formation and motion of dislocations. We show that the presence of dislocations in local equilibrium alone cannot explain charge transfer. However, under compression, their nonequilibrium dynamics that may involve direct excitation of phonons or conceivably electrons cannot be ruled out as a mechanism for charge transfer.

## Conclusions

Uniaxial pressing of MgO nanocube powders was found to produce characteristic spectroscopic property changes in the material that are indicative of charge separation effects at the surfaces and interfaces of the nanograins. In complementary theoretical calculations, we analyzed selected local contact configurations between MgO nanocubes and calculated the associated energetics upon external mechanical load. While reference experiments on light-induced charge separation, producing identical EPR and optical spectroscopic fingerprints, have revealed 4.6 eV as a minimum excitation energy for charge separation, the computational results indicate energies below this value.

In the field of tribocharging, this study provides for the first time molecular-level information on the pressure-induced separation and interfacial transfer of charge carriers on MgO grains. Since these processes can occur prior to the step of sintering, such insights serve as an important basis for a knowledge-based design and engineering of interfaces in compressed metal oxide nanoparticle powders. Related processing steps that trigger interesting interface chemistry can be performed at temperatures significantly below those typically required for the sintering of ceramics. Hence, so far unavailable mechanistic details will become accessible for the newly developed process of cold sintering.^49^ Moreover, the triboemission-induced generation of surface radicals offers a novel opportunity region to initiate radical polymerization reactions that can evolve in the confined space in between the grains, which may lead to nanoparticle–polymer composites of superior homogeneity and density.

## Supporting Information Available

Details about EPR powder simulations; additional EPR experiments performed; and complementary information comparing EPR results after compaction to those after UV excitation (PDF)
