# Supplementary material for: Rubbing Powders: Direct Spectroscopic Observation of Triboinduced Oxygen Radical Formation in MgO Nanocube Ensembles
Source: J Phys Chem C Nanomater Interfaces. 2021 Sep 29;125(40):22239–48. doi: 10.1021/acs.jpcc.1c05898 (PMC8521521; doi:10.1021/acs.jpcc.1c05898)
Supplement: Supplementary file 1 — jp1c05898_si_001.pdf [file jp1c05898_si_001.pdf]

Supporting Information

-

Rubbing Powders: Direct Spectroscopic Observation of Tribo-Induced Oxygen Radical Formation in MgO Nanocube Ensembles

Thomas Schwab[1], Daniel Thomele[1], Korbinian Aicher[1],

John W. C. Dunlop[1], Keith McKenna[2,] and Oliver Diwald[1,*]*

[1]Department of Chemistry and Physics of Materials, Paris-Lodron University Salzburg, Jakob-Haringer-Straße 2a, A-5020 Salzburg, Austria

[2]Department of Physics, University of York, Heslington, YO10 5DD York, United Kingdom

E-mail: oliver.diwald@plus.ac.at; keith.mckenna@york.ac.uk

- **Table of contents**

**EPR powder spectrum simulation**	
Table S1: Data of spin systems used during Easyspin simulations	2
**Additional EPR experiments**	
Figure S1: EPR spectrum acquisition of a powder compact after oxygen admission as well as after subsequent pumping to high vacuum (1)	3
Table S2: Corresponding mean g-values of Figure S1	4
Figure S2: EPR spectrum acquisition of a powder compact after oxygen admission as well as after subsequent pumping to high vacuum (2)	5
Table S3: Corresponding mean g-values of Figure S2	6
**Complementary information (Compaction vs. UV-excitation)**	
Figure S3: Complementary information comparing oxygen-related paramagnetic species after compaction and UV-excitation	7
Table S4: Corresponding mean g-values of Figure S3	7

Experimentally accessed EPR spectra were reproduced to the best of our knowledge with a minimum of paramagnetic spin centres and mean g-values that match well with those of the experiments to model the strongly broadened signal recorded during the experiment. The best fit was obtained by the superimposition of signals with characteristic g-values that are in the range of O^-, O_2^- and O_3^- species adsorbed on the MgO surface. The individual species were modeled by the superimposition of species of the same type (axial or rhombic) and by variations of the linewidth, their proportion to the overall signal and including contributions of unresolved hyperfine coupling. Details about mean g-values of the simulated spin systems are provided in Table S1*.*

Table S1: Mean g-values used within spectral analysis of the best fit, which also assumes contributions from unresolved hyperfine couplings (H_{Strain}) compared to those accessed within the compaction and UV-excitation experiments (compare Figure 4).

Powder compaction

Trapped holes O^-	Exp.		$g_\perp = 2.0321$ $g_\parallel = 1.9982$
	Sim.	(1)	$g_\perp = 2.0368$ $g_\parallel = 1.9984$
		(2)	$g_\perp = 2.0326$ $g_\parallel = 1.9984$
		(3)	$g_\perp = 2.0270$ $g_\parallel = 1.9984$
Superoxide anions O_2^-	Exp.		$g_{zz} = 2.0748$ $g_{yy} = 2.0042$ $g_{xx} = 1.9982$
	Sim.	(1)	$g_{zz} = 2.0780$ $g_{yy} = 2.0041$ $g_{xx} = 1.9984$
		(2)	$g_{zz} = 2.0729$ $g_{yy} = 2.0041$ $g_{xx} = 1.9984$
Ozonide anions O_3^-	Exp.		$g_{yy} = 2.0114$ $g_{zz} = 2.0084$ $g_{xx} = 1.9982$
	Sim.	(1)	$g_{yy} = 2.0115$ $g_{zz} = 2.0090$ $g_{xx} = 1.9984$
		(2)	$g_{yy} = 2.0115$ $g_{zz} = 2.0083$ $g_{xx} = 1.9984$

UV-excitation

Trapped holes O^-	Exp.		$g_\perp = 2.0306$ $g_\parallel = 1.9987$
	Sim.	(1)	$g_\perp = 2.0280$ $g_\parallel = 1.9987$
Superoxide anions O_2^-	Exp.		$g_{zz} = 2.0855$ $g_{yy} = 2.0050$ $g_{xx} = 1.9987$<br>$g_{zz} = 2.0693$
	Sim.	(1)	$g_{zz} = 2.0870$ $g_{yy} = 2.0044$ $g_{xx} = 1.9987$
		(2)	$g_{zz} = 2.0700$ $g_{yy} = 2.0068$ $g_{xx} = 1.9987$
		(3)	$g_{zz} = 2.0854$ $g_{yy} = 2.0041$ $g_{xx} = 1.9987$
Ozonide anions	Exp.		$g_{yy} = 2.0123$ $g_{zz} = 2.0086$ $g_{xx} = 1.9987$
	Sim.	(1)	$g_{yy} = 2.0123$ $g_{zz} = 2.0080$ $g_{xx} = 1.9987$

O_3^-		(2)	$g_{yy} = 2.0140$ $g_{zz} = 2.0096$ $g_{xx} = 1.9987$

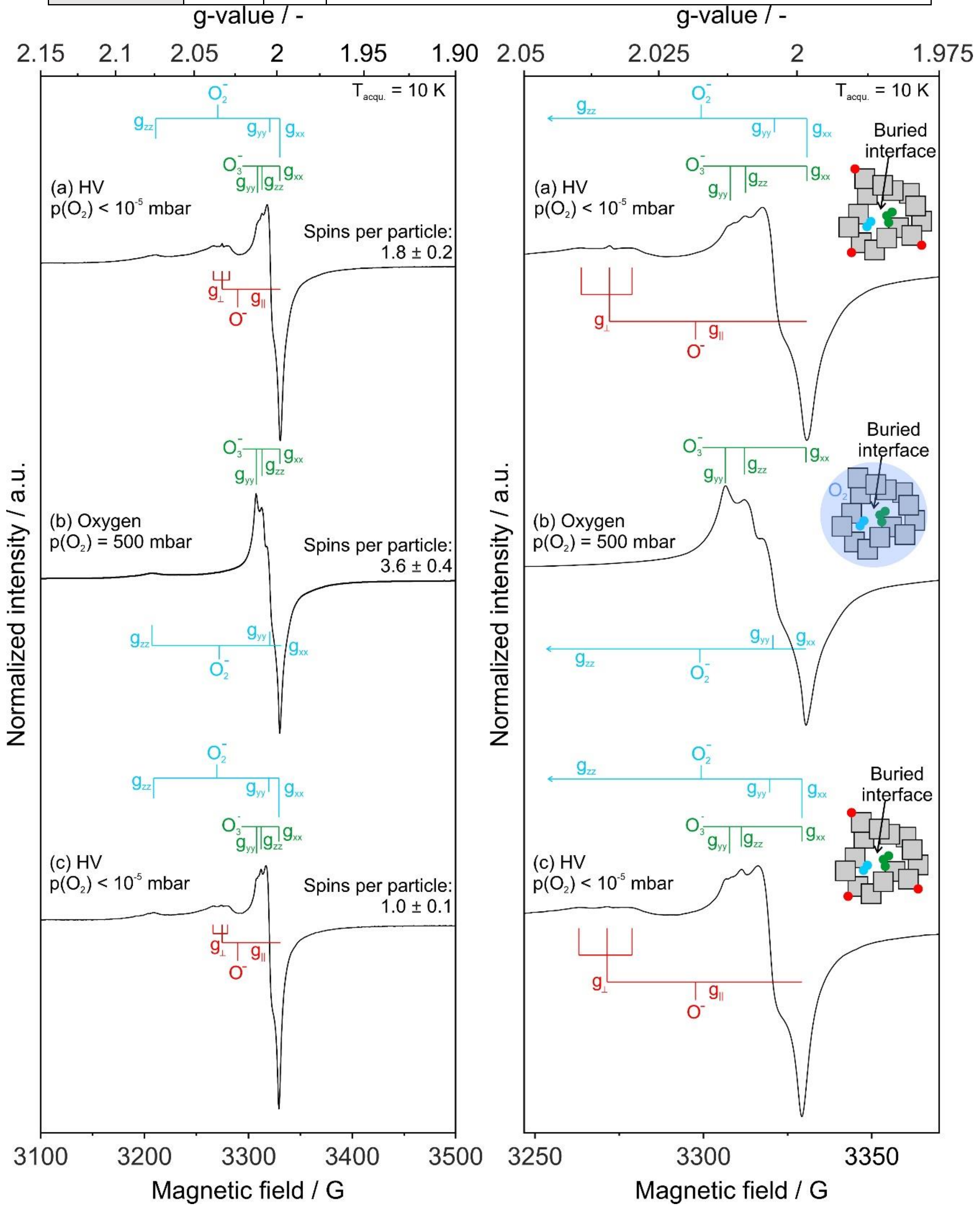

Figure S1: Wide magnetic field range (left) and oxygen radical related fingerprint region (right) electron paramagnetic resonance spectra of annealed MgO after uniaxial powder compaction (from top to bottom): (a) directly after compaction in HV, (b) in oxygen and (c) after subsequent pumping to HV at 10 K. (Note: Both oxygen admission and subsequent pumping were performed at 10 K. (Limited diffusion of O_2 but still electron transfer is possible)).

Table S2: EPR detected g-values of oxygen radicals at $T_{acqu.}$ = 10 K (Figure S1).

Trapped hole centers O^-	(a) Dynamic HV ($p(O_2)$ < 10^{-5} mbar)	$g_\perp = 2.0321$	$g_\parallel = 1.9982$	
	(b) Oxygen ($p(O_2)$ = 500 mbar)	–	–	
	(c) Dynamic HV ($p(O_2)$ < 10^{-5} mbar)	$g_\perp = 2.0325$	$g_\parallel = 1.9991$	
Superoxide anions O_2^-	(a) Dynamic HV (p < 10^{-5} mbar)	$g_{zz} = 2.0748$	$g_{yy} = 2.0042$	$g_{xx} = 1.9982$
	(b) Oxygen ($p(O_2)$ = 500 mbar)	$g_{zz} = 2.0764$	$g_{yy} = 2.0044$	$g_{xx} = 1.9989$
	(c) Dynamic HV ($p(O_2)$ < 10^{-5} mbar)	$g_{zz} = 2.0753$	$g_{yy} = 2.0049$	$g_{xx} = 1.9991$
Ozonide anions O_3^-	(a) Dynamic HV ($p(O_2)$ < 10^{-5} mbar)	$g_{yy} = 2.0114$	$g_{zz} = 2.0084$	$g_{xx} = 1.9982$
	(b) Oxygen ($p(O_2)$ = 500 mbar)	$g_{yy} = 2.0127$	$g_{zz} = 2.0087$	$g_{xx} = 1.9989$
	(c) Dynamic HV ($p(O_2)$ < 10^{-5} mbar)	$g_{yy} = 2.0116$	$g_{zz} = 2.0099$	$g_{xx} = 1.9991$

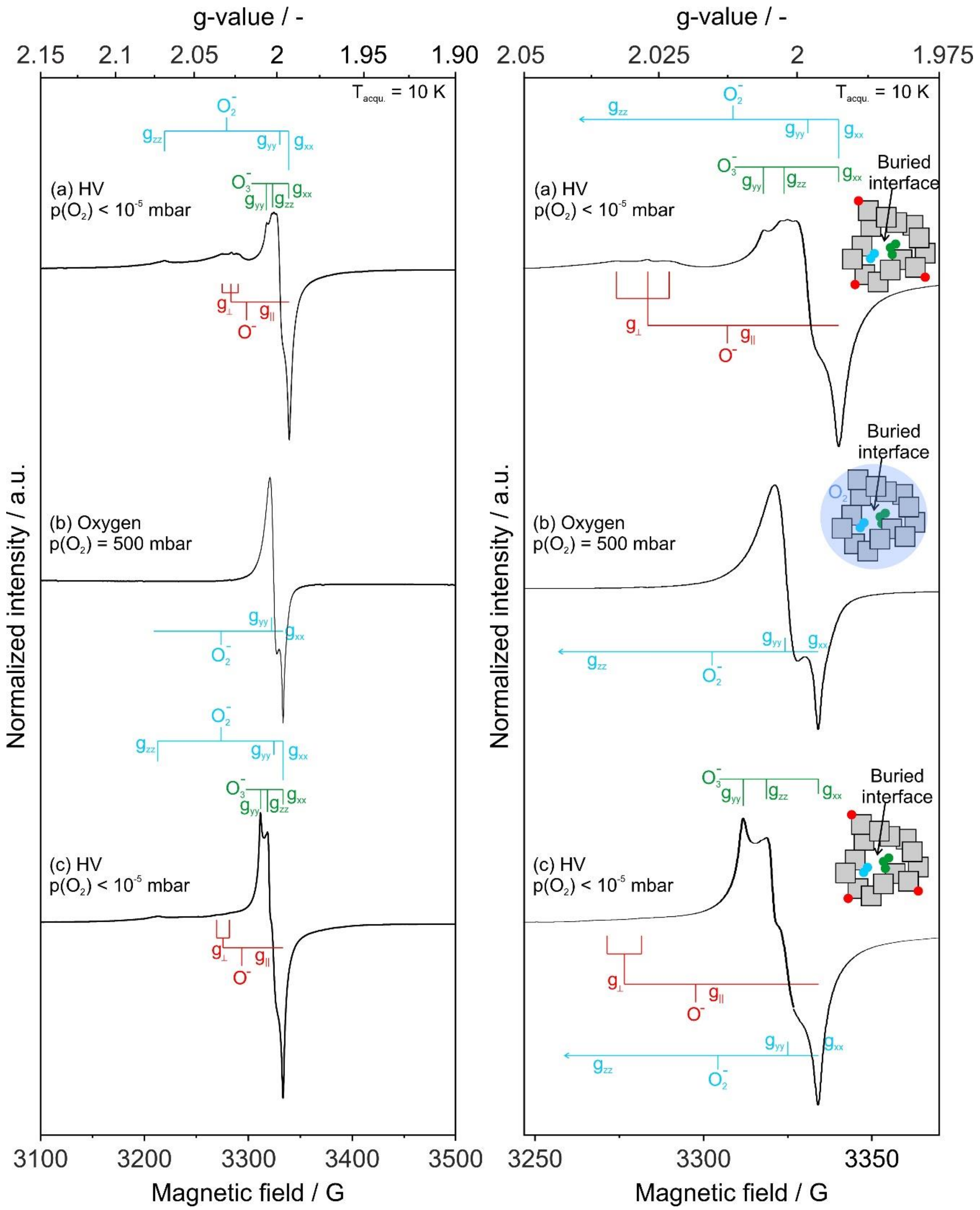

Figure S2: Wide magnetic field range (left) and oxygen radical related fingerprint region (right) electron paramagnetic resonance spectra of annealed MgO after uniaxial powder compaction (from top to bottom): (a) directly after compaction in HV, (b) in oxygen and (c) after subsequent pumping to HV at 10 K. (Note: Both oxygen admission and subsequent pumping were performed at room temperature (298 K). (Unlimited diffusion of O_2 leads to smearing out of the O^- signal)).

Table S3: EPR detected g-values of oxygen radicals at $T_{acqu.}$ = 10 K (Figure S2).

Trapped hole centers O^-	(a) Dynamic HV ($p(O_2) < 10^{-5}$ mbar)	$g_{\perp} = 2.0336$	$g_{\parallel} = 1.9996$	
	(b) Oxygen ($p(O_2)$ = 500 mbar)	–	–	
	(c) Dynamic HV ($p(O_2) < 10^{-5}$ mbar)	$g_{\perp} = 2.0338$	$g_{\parallel} = 1.9997$	
Superoxide anions O_2^-	(a) Dynamic HV ($p(O_2) < 10^{-5}$ mbar)	$g_{zz} = 2.0743$	$g_{yy} = 2.0046$	$g_{xx} = 1.9996$
	(b) Oxygen ($p(O_2)$ = 500 mbar)	$g_{zz} = -$	$g_{yy} = 2.0050$	$g_{xx} = 1.9996$
	(c) Dynamic HV ($p(O_2) < 10^{-5}$ mbar)	$g_{zz} = 2.0745$	$g_{yy} = 2.0055$	$g_{xx} = 1.9997$
Ozonide anions O_3^-	(a) Dynamic HV ($p(O_2) < 10^{-5}$ mbar)	$g_{yy} = 2.0127$	$g_{zz} = 2.0092$	$g_{xx} = 1.9996$
	(b) Oxygen ($p(O_2)$ = 500 mbar)	$g_{yy} = -$	$g_{zz} = -$	$g_{xx} = -$
	(c) Dynamic HV ($p(O_2) < 10^{-5}$ mbar)	$g_{yy} = 2.0129$	$g_{zz} = 2.0087$	$g_{xx} = 1.9997$

Complementary information to oxygen radicals observed on MgO nanocube compacts (compare to Figure 4)

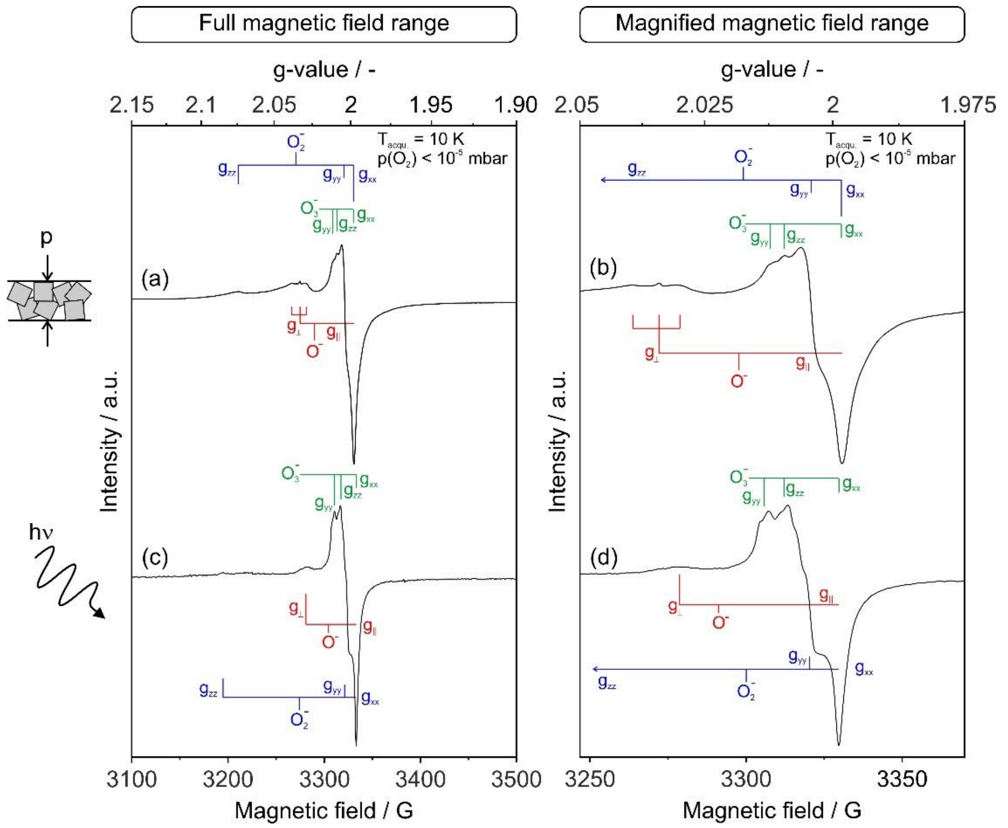

Figure S3: Full magnetic field range (left) containing the entire EPR signal after two different steps of radical production and the magnified magnetic field range (right). Spectra (a) and (b) were acquired on MgO nanocube powders after compaction at room temperature. Annealing of such a sample to 1173 K perfectly annihilates the paramagnetic species. Subsequent sample excitation with polychromatic UV light (c, d) produced an EPR fingerprint with identical g-parameters (Table S4).

Table S4: Comparison of g-parameters observed for radical species after powder compaction (Figure S3 a, b) and UV-excitation (Figure S3 c, d) of a previously EPR-silent powder compact.

Trapped holes O^-	(a, b) Powder compaction	$g_\perp = 2.0321$	$g_\parallel = 1.9982$	
	(c, d) UV-excitation	$g_\perp = 2.0306$	$g_\parallel = 1.9987$	
Superoxide anions O_2^-	(a, b) Powder compaction	$g_{zz} = 2.0748$	$g_{yy} = 2.0042$	$g_{xx} = 1.9982$
	(c, d) UV-excitation	$g_{zz} = 2.0855$<br>$g_{zz} = 2.0693$	$g_{yy} = 2.0050$	$g_{xx} = 1.9987$
Ozonide anions O_3^-	(a, b) Powder compaction	$g_{yy} = 2.0114$	$g_{zz} = 2.0084$	$g_{xx} = 1.9982$
	(c, d) UV-excitation	$g_{yy} = 2.0123$	$g_{zz} = 2.0086$	$g_{xx} = 1.9987$
